# Machine learning and mathematical modeling for comparative analysis of green-synthesized ZnO nanoparticles as seed nano-priming agents for linseed

**DOI:** 10.3389/fpls.2026.1827745

**Published:** 2026-05-11

**Authors:** Yusuf Şavşatlı, Muhammad Aasim, Ramazan Katırcı, Oktay Talaz, Muhammad Tanveer Altaf

**Affiliations:** 1 Department of Field Crops, Faculty of Agriculture, Recep Tayyip Erdoğan University, Rize, Pazar, Türkiye; 2 Department of Precision Agriculture and Agricultural Robots, Faculty of Agricultural Sciences and Technology, Sivas University of Science and Technology, Sivas, Türkiye; 3 Computer Engineering, Sivas University of Science and Technology, Sivas, Türkiye; 4 Department of Chemistry, Kamil Ozdag Faculty of Science, Kaamanoglu Mehmetbey University, Karaman, Türkiye

**Keywords:** green synthesis, linseed, machine learning, response surface methodology, seed priming, zinc oxide nanoparticles

## Abstract

Linseed (*Linum usitatissimum* L.) is a multipurpose crop valued for its oil, fiber, and nutraceutical properties, but micronutrient deficiencies and low nutrient-use efficiency often limit its productivity. Zinc is essential for plant physiological processes, yet conventional fertilization frequently suffers from poor bioavailability. Nanotechnology-based seed priming offers a promising approach to improve nutrient delivery and crop performance. This study evaluated green-synthesized zinc oxide nanoparticles (ZnO NPs) as seed priming agents in linseed, comparing their effects with those of chemically synthesized ZnO NPs and bulk ZnO. We hypothesized that biogenic ZnO nanoparticles would induce superior agronomic responses compared to chemical and bulk forms, exhibiting dose- and time-dependent (hormetic) effects that can be effectively captured using statistical and machine learning models. Seeds of cultivar ‘Yılmaz’ were primed with ZnO formulations at 25, 50, and 75 ppm for 1, 2, and 4 h. Experiments under field conditions assessed morphological and yield-related traits. ANOVA and response surface methodology (RSM) were used to analyze treatment effects, while machine learning models, including K-Nearest Neighbors (KNN), Random Forest (RF), Extra Trees, Support Vector Regression (SVR), Extreme Gradient Boosting (XGBoost), Categorical Boosting (CatBoost), and Multi-Layer Perceptron (MLP), modeled nonlinear relationships between nanoparticle treatments and plant traits. Biogenic ZnO nanoparticles significantly enhanced agronomic traits compared with chemical nanoparticles and bulk ZnO. Optimal responses were observed at moderate concentrations (25 ppm) and specific exposure durations, showing a hormetic effect. RSM indicated significant interactions among nanoparticle type, concentration, and priming duration. KNN achieved the highest predictive accuracy (R² = 0.84), and feature analysis identified nanoparticle concentration and exposure time as key factors. These results demonstrate that biogenic ZnO nanoparticle seed priming is a sustainable strategy to improve linseed productivity, highlighting the value of integrating experimental and data-driven approaches in crop research.

## 1 Introduction

Linseed (*Linum usitatissimum* L.) is one of the oldest cultivated crops, and it remains agronomically and economically significant due to its dual role as an oilseed and fiber crop (Zuk et al., 2015). Its seeds are rich in α-linolenic acid, lignans, proteins, and dietary fiber, making linseed increasingly important in functional foods, nutraceuticals, and health-oriented agricultural systems (Langyan and Kumar, 2024). In addition, linseed oil is widely used in industrial applications such as paints, varnishes, and biodegradable materials, while flax fiber contributes to sustainable textile and composite industries (Paliwal et al., 2023). Despite its broad utility, linseed productivity is often constrained by environmental stresses, suboptimal nutrient management, and micronutrient deficiencies, leading to unstable yields in many production regions.

Zinc (Zn) is an essential micronutrient involved in numerous physiological and biochemical processes, including enzyme activation, auxin metabolism, membrane stabilization, and antioxidant defense (Kolesnikov et al., 2025). Zinc deficiency is prevalent in agricultural soils worldwide and can significantly impair plant growth, branching, reproductive development, and seed yield (Hamzah Saleem et al., 2022). Conventional zinc fertilization strategies, such as soil and foliar applications of bulk zinc salts, often exhibit limited efficiency due to low solubility, soil fixation, and limited plant uptake (Dimkpa and Bindraban, 2016). These limitations highlight the need for innovative and sustainable approaches to enhance zinc bioavailability and nutrient use efficiency in crops such as linseed.

Nanotechnology offers promising solutions to address these challenges (El-Mogy et al., 2025; Monreal et al., 2016). Zinc oxide nanoparticles (ZnO NPs) possess unique physicochemical properties, including high surface area and enhanced reactivity, which may improve nutrient delivery and uptake compared to conventional bulk formulations (Al Jabri et al., 2022; Sheteiwy et al., 2021). Seed nanopriming, involving pre-sowing treatment with nanoparticle suspensions, has emerged as a targeted and resource-efficient strategy to enhance germination, early vigor, and subsequent plant performance (Yadav and Mathur, 2025; Dadlani, 2025). However, plant responses to nanoparticles are strongly influenced by particle characteristics, synthesis method, concentration, and exposure duration (Ravishankar et al., 2025; Iavicoli et al., 2018). While appropriate doses may stimulate growth and physiological activity, excessive levels can induce phytotoxic effects. Therefore, systematic evaluation and optimization of nanoparticle treatments are essential.

Traditional chemical synthesis of nanoparticles often involves hazardous reagents and energy-intensive processes, raising environmental and sustainability concerns. In contrast, green synthesis approaches utilize biological materials such as plant extracts or agro-industrial byproducts as reducing and stabilizing agents, offering eco-friendly and cost-effective alternatives (Nyandoro et al., 2025). Biogenic nanoparticles may also exhibit improved compatibility with plant systems due to natural surface functionalization (Senthamizh et al., 2025). Despite increasing interest in green nanotechnology, comparative studies assessing biogenically synthesized ZnO nanoparticles, chemically synthesized ZnO nanoparticles, and conventional bulk ZnO in linseed remain limited, particularly with respect to growth and yield-related traits.

Modern plant sciences experiments involve complex biological systems and are modulated by multiple interacting variables. The optimization of multiple factors can be done by using the design of experiments (DOE). This statistical technique enables researchers to design experimental variables or analyze post-experiment results by using multiple statistical tools to predict the best treatment level rather than a trial-and-error approach (Shina, 2022). Other advantages of DOE-based modeling include identifying key factors driving plant responses, understanding complex interactions, and optimization. Factorial regression analysis (FR), response surface regression (RSR), and Taguchi Design analysis are DOE-based statistical tools. RSR is used to model and analyze the impact of multiple experimental factors on one or more response variables (Katirci et al., 2021; Kisacik and Aasim, 2025). It identifies significant linear, quadratic, and interaction effects among variables, generates a polynomial equation to predict traits, and maps three-dimensional surface plots for optimizing two continuous variables (Ali and Aasim, 2024). The use of RSR in plant sciences has been prevalent in recent years for optimizing culture conditions for melon (Zhang et al., 2020), optimizing hormonal synthesis (Waday et al., 2022), optimizing basal nutrients for *in vitro* conservation of endangered plants (Bao et al., 2025), and *in vitro* propagation of sansevieria (Kohan-Baghkheirati et al., 2026), and aquatic Brazilian micro sword (Ali and Aasim, 2024).

Recent advances in machine learning have introduced powerful analytical techniques capable of modeling nonlinear relationships and high-dimensional data in agricultural systems (Waqas et al., 2025; Aasim et al., 2026; Altaf et al., 2026). Emerging quantum machine learning approaches further expand computational capacity by integrating principles of quantum computing with data-driven modeling (Katırcı et al., 2025). Although still in development, such techniques offer potential advantages for predictive modeling and optimization of multifactorial crop experiments.

This study aims to assess the potential of green-synthesized ZnO nanoparticles as seed priming agents for linseed, examining their comparative impact on agronomic performance relative to chemically synthesized ZnO nanoparticles and bulk ZnO. We postulate that biogenic ZnO nanoparticles will improve linseed growth and yield by enhancing nutrient uptake and stimulating growth, with effects that vary with nanoparticle concentration and exposure duration, exhibiting a hormetic response. Integrating green nanotechnology, statistical optimization, and advanced computational modeling provides a comprehensive strategy for sustainable crop improvement. The combined application of Taguchi design and quantum learning algorithms facilitates treatment optimization and predictive analysis, contributing to precision agriculture and improved micronutrient management in linseed production systems.

## 2 Materials and methods

### 2.1 Plant material and nanoparticle sources

Certified seeds of linseed (*Linum usitatissimum* L.) cultivar ‘Yılmaz’ were procured from the Black Sea Agricultural Research Institute (Turkey). Before experimentation, seeds were carefully screened to ensure uniformity in size, physical integrity, and germination capacity, thereby minimizing experimental variability and maintaining consistency across treatments. Three zinc-based treatments were evaluated in this study: (i) biogenically synthesized zinc oxide nanoparticles (ZnO-NP1), (ii) chemically synthesized zinc oxide nanoparticles (ZnO-NP2), and (iii) conventional bulk ZnO (analytical grade; molecular weight 81.39 g mol^-^¹). The inclusion of both nano-formulations and bulk ZnO allowed for a thorough comparison between green-synthesized nanoparticles, chemically synthesized nanoparticles, and their bulk ZnO counterpart. The experiment was designed to investigate the influence of nanoparticle type, concentration, and exposure duration on plant growth and yield-related traits.

### 2.2 Green synthesis of ZnO nanoparticles

Biogenic ZnO nanoparticles were synthesized via an eco-friendly approach utilizing sugar beet pulp (*Beta vulgaris* L.), procured from the sugar industry. Being an agro-industrial byproduct rich in reducing sugars and phenolic compounds, the phytochemical composition of the pulp, including reducing sugar content and total phenolics, was preliminarily characterized to confirm its suitability as a bio-reductant and stabilizing matrix. For nanoparticle synthesis, a zinc precursor salt (zinc acetate dihydrate or zinc nitrate hexahydrate) was dissolved in deionized water at a predetermined molarity. The prepared sugar beet pulp extract was gradually incorporated into the zinc solution under continuous magnetic stirring. The reaction mixture was maintained at controlled temperatures (ambient to 80 °C) and pH values ranging between 5.0 and 7.0 to facilitate the reduction of Zn²^+^ ions and subsequent nanoparticle formation. Nanoparticle generation was initially indicated by a visible change in solution coloration and further verified through UV-Visible spectrophotometric analysis. The synthesized ZnO nanoparticles were recovered by centrifugation, repeatedly washed with deionized water to eliminate residual organic impurities, and oven-dried before application. The crystallographic characteristics of the ZnO nanoparticles were examined using an X-ray diffractometer (Bruker D8 Advance) with Cu Kα radiation (λ = 1.5406 Å). The surface morphology of both commercial and biogenically synthesized nanoparticles was investigated through field emission scanning electron microscopy (FE-SEM; HITACHI SU5000, Japan), equipped with an energy-dispersive X-ray (EDX) analyzer to determine elemental composition. Fourier transform infrared (FT-IR) spectroscopy was performed using a Bruker Vertex ATR-FTIR spectrometer (Bruker Optics, USA) to identify the functional groups associated with the nanoparticle surfaces (**Supplementary Figure 1**).

### 2.3 Seed priming procedure

Seed priming treatments were conducted using aqueous suspensions of ZnO nanoparticles at concentrations of 25, 50, and 75 ppm. Seeds were immersed in the respective nanoparticle solutions for exposure durations of 1, 2, and 4 hours. A hydro primed control (distilled water) was included under identical temporal conditions. Following priming, seeds were surface-dried at room temperature under controlled laboratory conditions to restore their initial moisture content before sowing.

### 2.4 Experimental site and growth conditions

The study was carried out at the experimental area of the Department of Agriculture, Recep Tayyip Erdoğan University (41°10.668′ N, 40°54.018′ E; 65 m above sea level), Turkey. Seeds were initially cultivated under greenhouse conditions beginning April 11, 2025. A homogenized growth substrate was prepared consisting of 4/6 field soil, 1/6 peat, and 1/6 fine river sand to ensure adequate aeration and drainage. Eleven seeds were sown per pot at a uniform depth of approximately 2 cm. Soil moisture was maintained near field capacity through regular irrigation based on visual and tactile assessment of substrate moisture status. On May 31, 2025, all experimental units were transferred outdoors to continue growth under natural environmental conditions until physiological maturity.

### 2.5 Trait measurement and phenotypic evaluation

At full maturity, plants were carefully uprooted to avoid structural damage. Morphological, yield, and biomass-related traits were recorded, including Plant height (PH) (cm), Stem biomass per plant (SBP) (g) refers to the fibrous vegetative portion of the plant, specifically the biomass above the root system and below the reproductive (fruit-bearing) structures, Capsules/main panicle NCMP), Capsules/Plant (NCP), Seeds/capsule (NSC), Seeds weight/capsule (SWC) (mg/capsule), Seed yield per plant (SYP) (g/plant), Capsule length (CL) (mm), 1000 seeds weight.

### 2.6 Dataset description and preprocessing

The experimental dataset consists of experimental measurements related to flax cultivation under various NP treatments. The dataset serves as the foundation for a multi-output regression analysis aimed at predicting physiological and morphological plant traits. The dataset consists of 4 input variables (NP concentration, exposure time, repetition, and the specific type of NP) and 9 output variables (PH, SBP, NCMP, NCP, NSC, SWC, SYP, CL, and 1000 seed weight). Before model training, data preprocessing was performed to ensure compatibility with ML algorithms. The categorical feature NP was transformed using One-Hot Encoding, converting the qualitative data into a binary matrix format (Okada et al., 2019). For distance-based and gradient-based algorithms, continuous input variables were normalized using Standard Scaler to standardize features to a mean of 0 and a standard deviation of 1, preventing features with larger magnitudes from dominating the objective function (Ali et al., 2014).

### 2.7 Machine learning algorithms

A Direct Multi-Output Regression strategy was used to address the prediction of multiple physiological traits, as this approach decomposes the problem into separate regression tasks instead of training a single model to predict all targets at once. Specifically, an independent model was trained for each of the 9 output variables, as this allows the system to learn specific feature interactions for each trait individually, while parallel processing was employed to execute these training tasks. Seven distinct ML algorithms were used to evaluate the predictive performance, as each ML algorithm represents a different learning paradigm being used. Random forest (RF) is an ensemble learning method that constructs a multitude of decision trees during training and outputs the average prediction of the individual trees to reduce overfitting (Pavlov, 2019). ExtraTrees (ET) is an extremely randomized tree method that introduces additional randomness in split selection, often reducing the variance further than Random Forest (RF). Extreme Gradient Boosting (XGBoost) is a scalable and efficient implementation of gradient boosting that utilizes a sparsity-aware algorithm and tree learning (Kharwar and Thakor, 2022). Categorical Boosting (CatBoost) is a gradient boosting algorithm specifically designed to handle categorical data effectively and reduce prediction shift (Prokhorenkova et al., 2018). K-nearest neighbor (KNN) is a non-parametric method that predicts values based on the average of the k closest training examples in the feature space (Katırcı et al., 2021). A Multilayer Perceptron (MLP) is a feedforward artificial neural network consisting of fully connected layers, capable of learning non-linear relationships through backpropagation (Hesami et al., 2021). Support vector regressor (SVR) is a kernel-based method that finds a hyperplane in a high-dimensional space to minimize error while maintaining a specified margin of tolerance (Katirci et al., 2021).

### 2.8 Hyperparameter optimization and model training

A Hyperparameter Optimization process was conducted using Grid Search logic to maximize the model performance, and a specific grid of hyperparameters was defined for each algorithm (**Table 1**). A 5-Fold CV was utilized for optimization. The dataset was randomly partitioned into 5 subsamples; a single subsample was retained as the validation data for testing the model, and the remaining 4 subsamples were used as training data. This process was repeated 5 times, providing an estimate of model performance and minimizing selection bias. Hyperparameter optimization was performed by targeting the highest R^2^ scores across all trait-output pairs. For each algorithm, a specific search space, including parameters, such as the number of estimators, learning rates, and tree depths was defined and rigorously explored.

**Table 1:** Defined hyperparameter search space and grids used for the optimization of different machine learning algorithms.

Model	Hyperparameter search space (grid)
KNN	n_neighbors: [3, 5, 7, 9], weights: [‘uniform’, ‘distance’], metric: [‘euclidean’, ‘manhattan’]
RF	n_estimators: [50, 100, 200], max_depth: [None, 10, 20], min_samples_split: [2, 5, 10]
ET	n_estimators: [50, 100, 200], max_depth: [None, 10, 20], min_samples_split: [2, 5, 10]
XGBoost	n_estimators: [100, 200], learning_rate: [0.01, 0.1, 0.2], max_depth: [3, 5, 7]
CatBoost	iterations: [100, 200], learning_rate: [0.01, 0.1], depth: [4, 6, 8], logging_level: [‘Silent’]
MLP	hidden_layer_sizes: [(50), (100), (50, 50)], activation: [‘relu’, ‘tanh’], solver: [‘adam’, ‘sgd’]
SVR	kernel: [‘rbf’, ‘poly’, ‘sigmoid’], C: [0.1, 1, 10], epsilon: [0.01, 0.1]

KNN, K-Nearest Neighbors; RF, Random Forest; ET, Extremely Randomized Trees; XGBoost, Extreme Gradient Boosting; CatBoost, Categorical Boosting; MLP, Multilayer Perceptron; SVR, Support Vector Regression.

### 2.9 Performance evaluation metrics

The performance of the developed models was evaluated using a comprehensive set of statistical metrics to assess accuracy, error magnitude, and variance explanation. Coefficient of determination (R^2^) was used as the primary metric to measure the proportion of variance in the dependent variable from the independent variables (Equation 1). Mean squared error (MSE) was used to calculate the average squared difference between the estimated values and the actual values (Equation 2). Root mean squared error (RMSE) is used to measure the standard deviation of the prediction errors (Equation 3). Mean absolute error (MAE) was used to compute the average magnitude of errors in a set of predictions, without considering direction (Equation 4). Mean absolute percentage error (MAPE) expresses accuracy as a percentage of the error compared to the actual values (Equation 5). Explained Variance Score (EVS) is used to measure the discrepancy between a model and actual data (Equation 6).

(1) $$R^{2}=1-\;\frac{\sum_{i=1}^{n}{\left(Y_{i}-{\hat{Y}}_{i}\right)}^{2}}{\sum_{i=1}^{n}{\left(Y_{i}-\tilde{Y}\right)}^{2}}$$

(2) $$MSE=\frac{1}{n}\sum_{i=1}^{n}{\left(y_{i}-\hat{y_{i}}\right)}^{2}$$

(3) $$RMSE=\;\sqrt{\frac{1}{n}\;\sum_{i=1}^{n}{\left(Y_{i}-{\hat{Y}}_{i}\right)}^{2}}\;$$

(4) $$MAE=\frac{1}{n}\sum_{i=1}^{n}|y_{i}-\hat{y_{i}}|$$

(5) $$MAPE=\frac{100\%}{n}\sum_{i=1}^{n}|\frac{y_{i}-\hat{y_{i}}}{y_{i}}|$$

(6) $$\text{EVS}=1-(\text{Var}(\text{y}-{\text{y}}^{⌃})/\text{Var}(\text{y}))$$

### 2.10 Feature importance analysis

To mitigate overfitting and enhance model generalizability, a 5-fold CV procedure during training was applied. Experimental replicates were incorporated through the inclusion of the repetition variables, and its contribution was also evaluated using permutation feature importance, confirming the treatment factors remained the primary determinants of model predictions.

### 2.11 Statistical analysis

In this study, three input variables (NP type, concentration, and exposure time) were used to analyze the results for 9 agronomic (growth- and yield-related) linseed traits. The experiment was replicated thrice, after which the data were computed and analyzed. The results of experimental data were analyzed by two different statistical approaches: traditional one-way and two-way ANOVA (analysis of variance) analysis, and a modern DOE-based statistical tool using RSR ANOVA. The Minitab 21.0 statistical software program was used, and the difference between means was computed by the Tukey test for traditional ANOVA. Whereas JAPS (0.95.4.0) statistical software program was used for RSR analysis and for constructing the Pareto chart and normal plots. To construct a 3-D surface plot for the individual NP type, the design expert (DX) statistical software program was used to get a more sophisticated visualization for optimizing two continuous factors (concentration and exposure time).

The entire analytical framework was implemented in the Python programming language. Key libraries used included Scikit-learn for model implementation and preprocessing, XGBoost and CatBoost for gradient boosting algorithms, Pandas and NumPy for data manipulation, and Matplotlib/Seaborn for data visualization. All computations were executed in a parallelized environment to handle the multi-output complexity efficiently.

## 3 Results

### 3.1 ANOVA analysis of agronomic traits

One-way ANOVA analysis of individual input factors on the agronomic traits is presented in **Supplementary Table 1**. All individual input variables exhibited a statistically significant but variable impact on agronomic traits. Impact of ZnONPs resulted in statistically significant impact on plant height (p0.01, stem biomass (p0.05), and number of seeds per capsule (p0.05). Pant height and number of seeds per capsule were maximum from B-ZnO-2 NPs, while stem biomass was high from the control treatment (1.927 g) (**Supplementary Table 1**). The results of concentration revealed a significant impact only for seeds per capsule and found more than the control (**Supplementary Table 1**), with a maximum of 25 mg/L treatment. Whereas treatment time statistically regulated the plant height only, and 4h of priming time yielded the maximum of 75.55 cm (**Supplementary Table 1**).

The results of the interaction of all individual factors (NPs × Conc. × Time) were totally different, and statistically significant at p0.01 (plant height, number of capsules per main panicle), and p0.05 (stem biomass per plant, and capsules per plant). Whereas capsule length was recorded as insignificant (**Table 2**). The highest plant height (81.63 ± 7,01) and technical length (58.67 ± 7.36) were recorded from the combination of B-ZnO-2 × 25 mg × 4h. Whereas interaction of B-ZnO-1 × 25 mg × 2h yielded the maximum stem biomass (2.006 ± 0.128) and capsules per plant (36.57 ± 4.23). The maximum seeds per capsule were computed from the nanopriming of B-ZnO-2 × 75 mg × 4h, and plant height by using ZnO × 25 mg × 1h. Two different interactions (B-ZnO-2 × 25 mg × 2h and B-ZnO-1 × 75 mg × 2h) were found to be optimum for the 1000 seed weight. Seed yield per plant was highly significant and regulated by 25 mg concentration for all three ZnO NPs.

**Table 2:** Interactive effect of ZnO nanoparticles × concentration × treatment time on agronomic traits of linseed.

Interaction	Plant height (cm)**	Stem biomass/plant (g) *	No. of capsules ın the maın**	No. of capsules/plant**	No. of seeds/capsule*	Seeds per capsule**	Seed yield g/plant)**	Capsule length (mm)ns	1000 seeds weight**
25-ZnO-1	68,53 ± 2,14b	1.277 ± 0.063ıjk	23.43 ± 1.629abcd	29.70 ± 1.73abcd	8.700 ± 0.361ab	60.20 ± 6.49a**	1.143 ± 0.050abcd	6.880 ± 0.316	5.807 ± 0.132abc
25-ZnO-2	76,50 ± 8,21ab	1.879 ± 0.119abcd	20.23 ± 2.66abcd	32.53 ± 3.53abcd	9.000 ± 0.458ab	52.71 ± 1.18ab	1.296 ± 0.016a**	7.126 ± 0.170	5.518 ± 0.098abcd
25-ZnO-4	70,77 ± 4,01ab	1.482 ± 0.126efghıjk	20.00 ± 2.36abcd	29.00 ± 4.86abcd	7.800 ± 1.114ab	47.12 ± 4.65bc	0.983 ± 0.029cdefgh	7.280 ± 0.585	5.411 ± 0.163abcd
50-ZnO-1	73,93 ± 3,87ab	1.588 ± 0.156defgh	20.90 ± 1.85abcd	30.67 ± 1.74abcd	8.067 ± 0.252ab	47.46 ± 1.96bc	0.930 ± 0.046efghı	6.943 ± 0.286	5.336 ± 0.120 bcd
50-ZnO-2	70,03 ± 2,11ab	1.315 ± 0.008hıjk	20.57 ± 1.504abcd	27.57 ± 4.78abcd	8.400 ± 0.624ab	44.69 ± 6.02bcdef	0.773 ± 0.016ı	7.237 ± 0.274	5.239 ± 0.088d
50-ZnO-4	75,10 ± 2,69ab	1.435 ± 0.063fghıjk	23.10 ± 5.03abcd	27.20 ± 4.00abcd	7.633 ± 0.493ab	36.49 ± 2.65ef	0.897 ± 0.021ghı	7.197 ± 0.402	5.324 ± 0.095 bcd
75-ZnO-1	69,13 ± 3,76ab	1.686 ± 0.125bcdef	17.23 ± 1.079cd	29.47 ± 4.90abcd	7.933 ± 0.493ab	42.17 ± 1.31cdef	1.133 ± 0.032abcde	7.220 ± 0.340	5.460 ± 0.028abcd
75-ZnO-2	72,40 ± 0,20ab	1.431 ± 0.026fghıjk	20.57 ± 4.13abcd	27.87 ± 3.88abcd	7.933 ± 0.950ab	36.90 ± 3.69def	0.890 ± 0.058ghı	7.340 ± 0.305	5.188 ± 0.011d
75-ZnO-4	70,63 ± 3,94ab	1.522 ± 0.060efghıj	20.67 ± 0.65abcd	26.90 ± 3.48abcd	7.733 ± 0.473ab	40.67 ± 1.86cdef	1.030 ± 0.063bcdefg	7.570 ± 0.082	5.305 ± 0.140cd
25-ZnO-1-1	74,73 ± 2,87ab	1.538 ± 0.074efghı	19.10 ± 3.15abcd	27.33 ± 3.17abcd	8.167 ± 0.351ab	45.52 ± 1.39bcde	0.897 ± 0.062ghı	7.367 ± 0.312	5.294 ± 0.134cd
25-ZnO-1-2	71,80 ± 4,59ab	2.006 ± 0.128a	23.43 ± 0.23abcd	36.57 ± 4.23a**	7.267 ± 0.503ab	43.27 ± 1.78bcdef	1.243 ± 0.085a**	7.293 ± 0.045	5.434 ± 0.064abcd
25-ZnO-1-4	71,27 ± 5,36ab	1.245 ± 0.033jk	21.20 ± 3.57abcd	24.23 ± 2.16 bcd	8.533 ± 0.451ab	45.46 ± 0.95 bcde	0.920 ± 0.039fghı	7.260 ± 0.368	5.482 ± 0.422abcd
50-ZnO-1-1	73,13 ± 2,87ab	1.756 ± 0.161abcde	25.43 ± 1.026ab	35.43 ± 3.20a**	7.767 ± 0.850ab	40.93 ± 0.62cdef	1.210 ± 0.044ab	6.820 ± 0.201	5.578 ± 0.345abcd
50-ZnO-1-2	73,90 ± 3,24ab	1.661 ± 0.102cdefgh	23.47 ± 3.66abcd	31.90 ± 3.44abcd	7.800 ± 0.586ab	44.93 ± 2.09bcdef	1.170 ± 0.074abcd	7.347 ± 0.297	5.533 ± 0.043abcd
50-ZnO-1-4	77,30 ± 3,60ab	1.520 ± 0.100efghıj	24.33 ± 1.88abc	30.20 ± 3.00abcd	7.467 ± 0.458ab	42.86 ± 4.59bcdef	1.187 ± 0.128abc	6.890 ± 0.141	5.655 ± 0.194abcd
75-ZnO-1-1	70,60 ± 1,80ab	1.493 ± 0.043efghıjk	22.47 ± 4.29abcd	31.80 ± 1.01abcd	0.231 ± 0.635ab	46.90 ± 1.83bc	1.173 ± 0.059abcd	7.300 ± 0.230	5.567± 0.02abcd
75-ZnO-1-2	72,23 ± 4,62ab	1.203 ± 0.034k	18.80 ± 3.15bcd	26.63 ± 3.06abcd	6.933 ± 0.681b	35.08 ± 0.66f	0.830 ± 0.058ghı	7.070 ± 0.442	5.585 ± 0.077abcd
75-ZnO-1-4	75,33 ± 4,05ab	1.875 ± 0.128abcd	19.33 ± 2.37abcd	30.20 ± 1.85abcd	7.733 ± 0.416ab	40.42 ± 2.98cdef	1.117 ± 0.114abcdef	7.513 ± 0.346	5.891 ± 0.215a**
25-ZnO-2-1	68,30 ± 4,22b	1.370 ± 0.047ghıjk	16.20 ± 1.015d	22.33 ± 2.08d	8.833 ± 0.702ab	42.74 ± 2.86bcdef	0.880 ± 0.022ghı	7.453 ± 0.285	5.425 ± 0.148abcd
25-ZnO-2-2	75,90 ± 2,84	1.707 ± 0.117bcdef	26.67 ± 3.55a**	34.43 ± 3.38ab	7.667 ± 1.021ab	46.55 ± 2.60bcd	1.247 ± 0.063a	7.353 ± 0.224	5.890 ± 0.150a**
25-ZnO-2-4	81,63 ± 7,01a	1.439 ± 0.126fghıjk	22.90 ± 1.587abcd	24.77 ± 2.04 bcd	8.767 ± 0.208ab	52.35 ± 2.64ab	0.977 ± 0.051defghı	6.847 ± 0.456	5.869 ± 0.117ab
50-ZnO-2-1	73,80 ± 2,86ab	1.749 ± 0.088abcde	19.90 ± 2.71abcd	30.90 ± 4.51cd	8.167 ± 0.929ab	42.13 ± 2.38 cdef	1.107 ± 0.058abcdef	7.536 ± 0.072	5.497 ± 0.379abcd
50-ZnO-2-2	77,87 ± 3,71ab	1.215 ± 0.106k	21.77 ± 1.079abcd	22.77 ± 2.04abcd	8.367 ± 0.635ab	49.107 ± 1.67bc	0.797 ± 0.054hı	7.300 ± 0.225	5.588 ± 0.058abcd
50-ZnO-2-4	78,93 ± 4,37ab	1.632 ± 0.018defg	23.57 ± 0.513abcd	32.43 ± 5.22abcd	7.767 ± 0.306ab	44.75 ± 2.84bcdef	1.186 ± 0.088abc	7.213 ± 0.169	5.589 ± 0.077abcd
75-ZnO-2-1	73,17 ± 2,64ab	1.681 ± 0.077bcdef	23.23 ± 0.924abcd	31.23 ± 0.92abcd	7.533 ± 1.401ab	44.94 ± 7.36bcdef	1.177 ± 0.016abcd	7.500 ± 0.204	5.56 ± 0.1311abcd
75-ZnO-2-2	78,30 ± 5,10ab	1.965 ± 0.041ab	23.63 ± 0.577abcd	33.43 ± 2.87ab	8.033 ± 0.208ab	41.327 ± 0.83cdef	1.077 ± 0.083cdefg	7.390 ± 0.395	5.333 ± 0.130 bcd
75-ZnO-2-4	78,37 ± 3,57ab	1.553 ± 0.049efghı	20.20 ± 0.173abcd	28.67 ± 4.38abcd	9.033 ± 0.651a*	44.23 ± 0.61bcdef	0.979 ± 0.070defgh	7.327 ± 0.280	5.367 ± 0.27 abcd
Control	74,63 ± 2,91ab	1.927 ± 0.027abc	23.86 ± 1.504abcd	33.20 ± 1.015abc	7.967 ± 0.416ab	39.82 ± 2.15cdef	1.174 ± 0.095abcd	7.223 ± 0.357	5.565 ± 0.04 abcd

**Significant at p < 0.01, *Significant at p < 0.05, ns, nonsignificant.

### 3.2 Response surface methodology analysis

**Figure 1:**
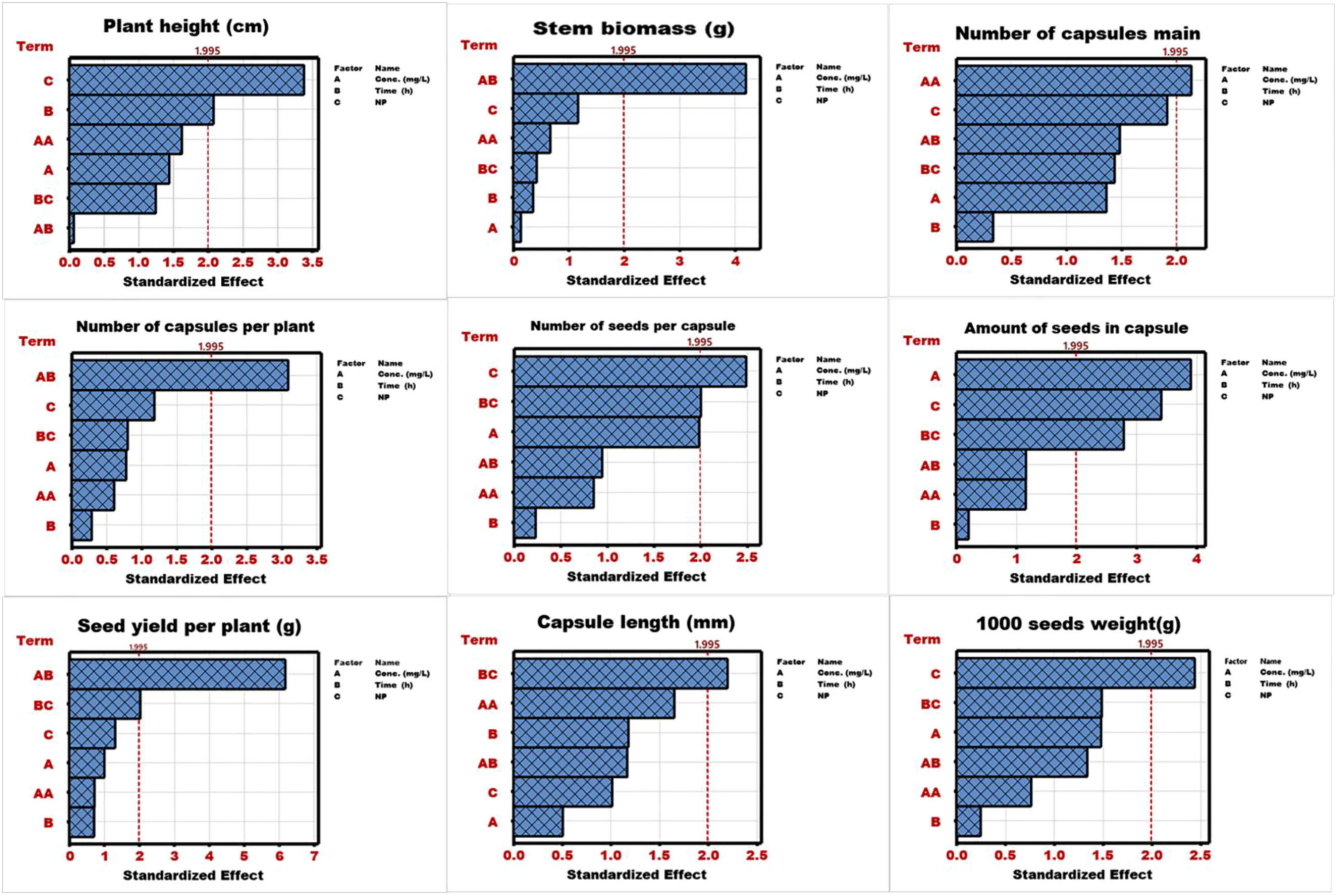
Pareto chart analysis of linseed studied traits under nanoparticle priming.

The ANOVA analysis of the RSM model is presented in **Supplementary Table 2**, which shows the variable responses to the predictors and indicates statistical significance at p0.01 and p0.05. The model’s significance was recorded for plant height, stem biomass per plant, seeds per capsule, and seed yield per plant, which were statistically significant at p0.01. whereas the number of seeds per capsule, capsule length, and 1000 seed weight were computed statistically significant at p0.05. The RSM-based ANOVA analysis was further used to analyze the results using a Pareto chart and a normal plot. Results of the Pareto chart are presented in **Figure 1**, and all response variables exhibited different responses to the predictors. The predictor factor in bold refers to the statistically significant impact of RSM analysis. The stacking order was recorded as C-B-AA-A-BC-AB for pant height, AB-C-AA-BC-B-A for stem biomass, AA-C-AB-BC-A-B for number of capsules, AB-C-BC-A-AA-B for capsules per plant, C-BC-A-AB-AA-B for number of seeds per capsule, A-C-BC-AB-AA-B for seeds per capsule, AB-BC-C-A-AA-B for seed yield per plant, BC-AA-B-AB-C-A for capsule length, and C-BC-A-AB-AA-B for 1000 seeds weight. In all these responses, at least one predictor was found significant. Whereas some response variables were computed statistically insignificant, and the following order was recorded as AA-AB-B-A-BC-C branches per plant, AB-BC-B-AA-A-C for fruiting zone length, AA-C-A-BC-B-AB for capsule diameter, AB-C-AA-A-B-BC for root weight, and AB-A-AA-BC-C-B for stem diameter.

**Figure 2:**
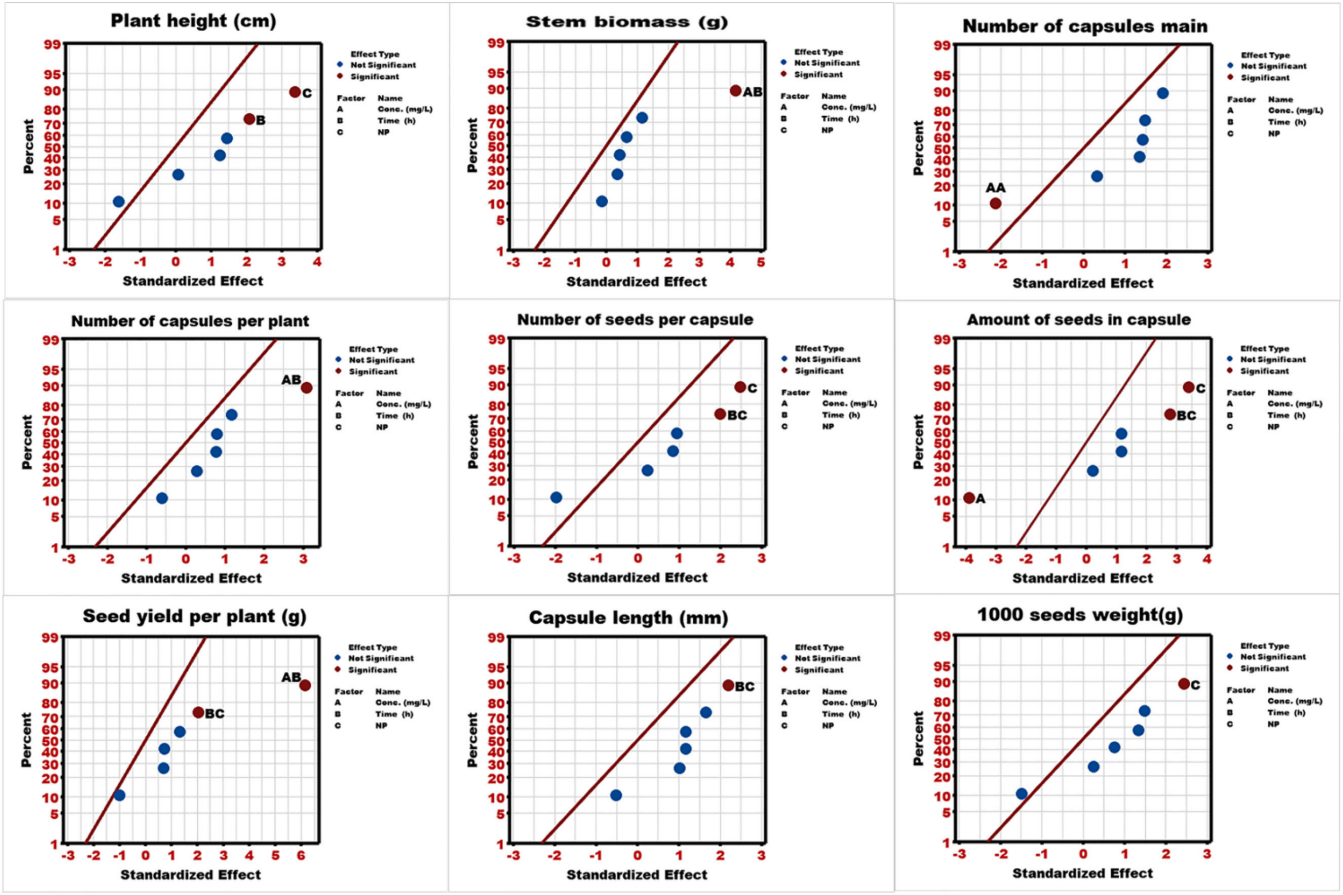
Normal plot analysis of linseed studied traits under nanoparticle priming.

The results of the Pareto chart were evaluated with the normal plot analysis, and most of the predictors were placed on the right side of the standard line, indicating the proportional impact (**Figure 2**). Considering significant predictors, AA for the number of capsules and A for seeds per capsule were placed on the left side, indicating the negative impact of increased concentration on their respective responses.

### 3.3 Correlation analysis of input and output variables

**Figure 3:**
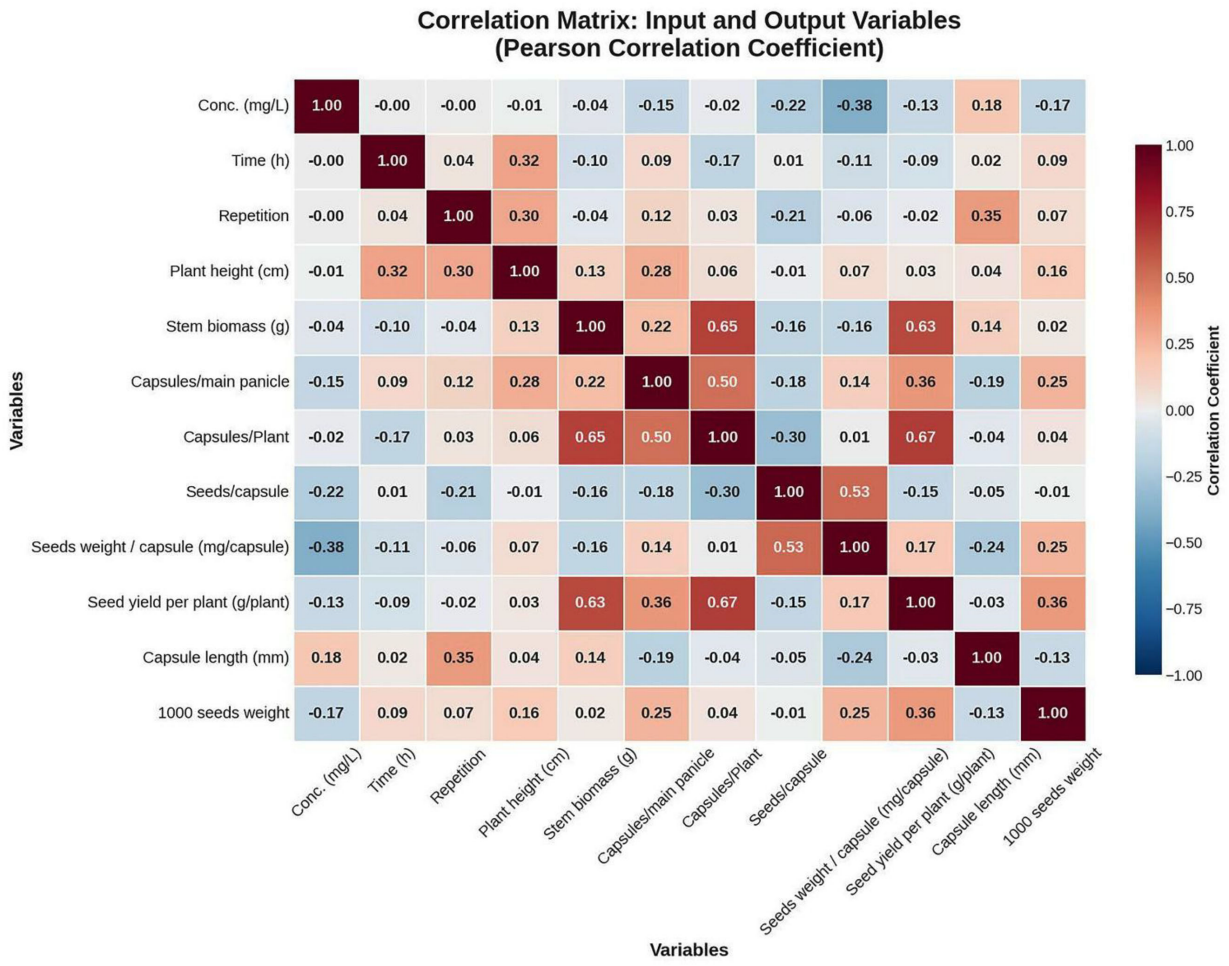
Correlation matrix displaying the associations among input features and flax morphological output variables.

A Pearson correlation analysis was conducted (**Figure 3**) to investigate the linear associations between experimental treatments and physiological traits, as well as the interdependencies among the response variables. A strong positive correlation was observed between SYP and CP, as well as between WP and CP. These suggest that plants with greater vegetative biomass and prolific capsule formation are primary determinants of overall SY. While SWC had a moderate positive correlation with SC. This suggests that capsules containing more seed tend to have a higher SW. While concentration showed a negative correlation with SWC and SYP, indicating that higher NP concentrations may indıce stree responses that inhibit seed development. Conversely, ET displayed a positive correlation with PH, consistent with expected growth dynamics over time. The presence of multicollinearity among the target variables supports the rationale for employing a Multi-Output Regression Strategy. By treating these correlated outputs as distinct but related tasks, the ML models can leverage these underlying patterns to enhance predictive accuracy, as shown by the superior performance of the KNN model in capturing these local data structures.

### 3.4 Machine learning modeling analysis

The overall predictive performance of the ML algorithms was evaluated using nine physiological and morphological traits of flax is presented in **Table 3**. Results demonstrated the best performance by the KNN model, with an R^2^ score of 0.8417 and an MSE score of 1.1108. These results suggest the relationship between NPs and plant traits is well-captured by the local neighborhood averaging approach of KNN. It was followed by ET and RF models with an R^2^ score of 0.681 and 0.654, respectively. Whereas XGBoost, CatBoost, and MLP demonstrated moderate performance, while SVR yielded the lowest performance with an R^2^ score of 0.271. Results suggest that default kernel functions failed to map out the non-linear complexities and demand extensive kernel tuning for better prediction.

**Figure 4:**
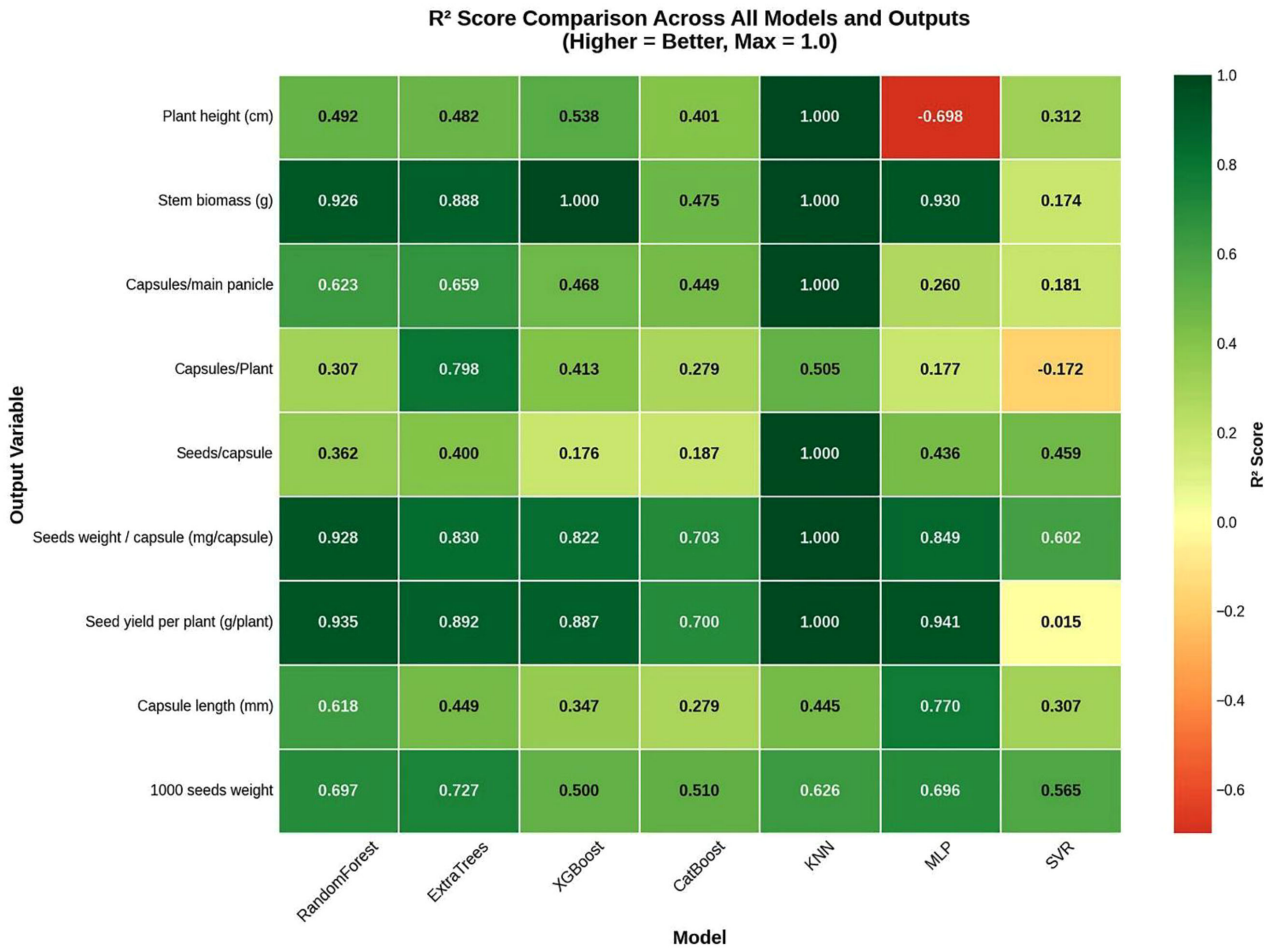
Heatmap of coefficient of determination scores across all models and output variables. Darker green indicates higher predictive accuracy.

**Figure 5:**
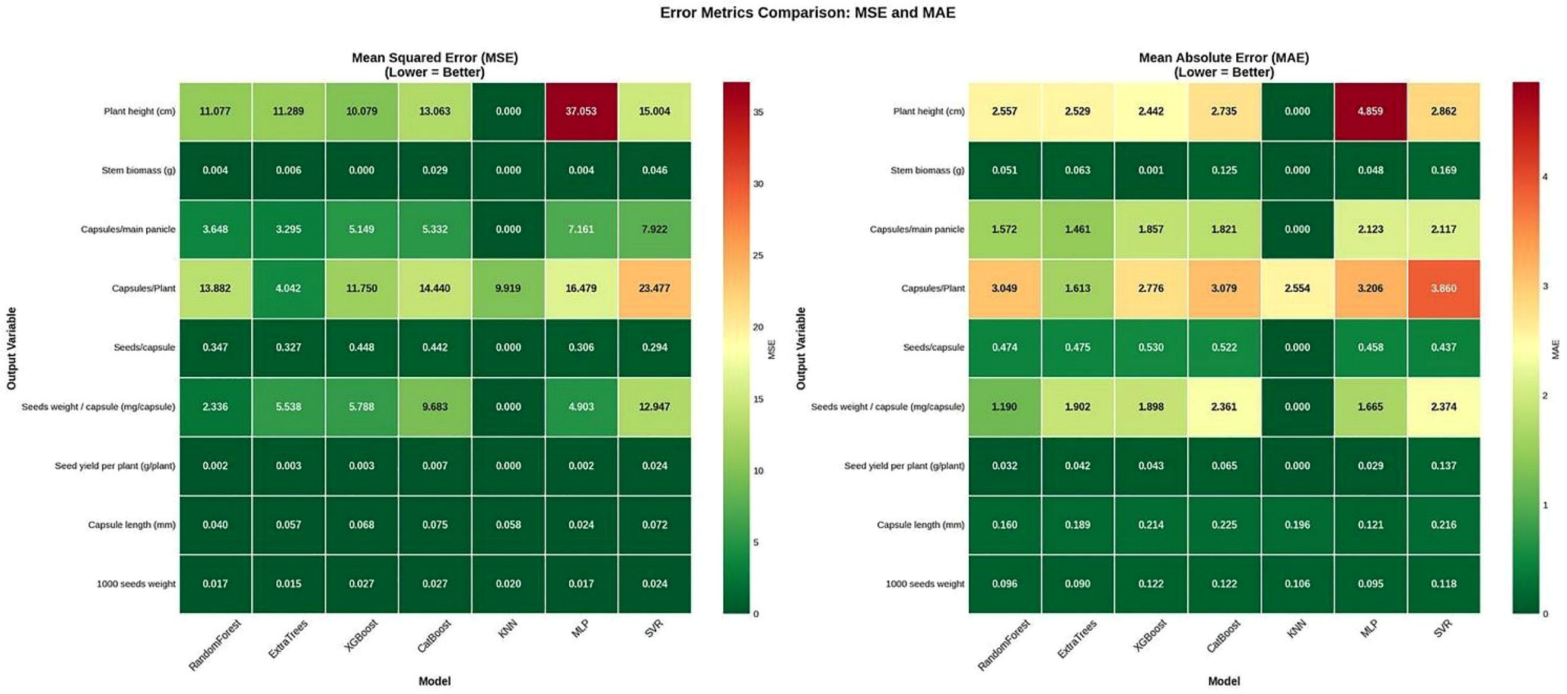
heatmaps displaying mean squared error and mean absolute error for each model-output pair. Darker green indicates lower error rates.

**Figure 4** illustrates the R^2^ scores for each model-output combination in a heatmap format, allowing for a detailed visual assessment of model strengths. The KNN model showed exceptional accuracy of R^2^ for certain traits such as PH, SBP, and SYP, suggesting a strong correlation between the input features and specific growth parameters. The lowest prediction with an R^2^ score for CP was recorded for the KNN model. Whereas CL exhibited a reasonable prediction ability with an R^2^ score ranging from 0.44 to 0.77, indicating that the trait was affected by extraneous environmental factors. To validate the reliability of the predictions, MSE and MAE were analyzed, as presented in **Figure 5**. KNN maintained the lowest error rates across the outputs, such as Seed yield per plot. KNN achieved a negligible error, whereas MLP and SVR exhibited higher MSE values. The discrepancy between RMSE and MAE across models suggests that while tree-based models are generally stable, they are occasionally prone to larger outlier errors compared to KNN in this specific experimental setup.

### 3.5 Optimal model selection per trait

From these results, KNN performed the best as shown in **Table 3**, and recommended a model for each specific output variable based on the maximization of R^2^ and the minimization of MSE. KNN was seen as the primary recommendation for most of the traits, while MLP showed a competitive performance for CL and SBP, outperforming the TB models in those traits. This highlights the importance of the multi-model approach, for practical applications aiming to optimize CL specifically, a neural network approach might be preferable over ET, despite the latter’s better average performance.

**Table 3:** Comprehensive summary of model performance metrics averaged across all nine output variables, sorted by Mean R^2^.

Model	R^2^ Mean	R^2^ Std	MSE Mean	RMSE Mean	MAE Mean	MAPE Mean (%)	Exp. Var. Mean
KNN	0.8417	0.2280	5.1930	0.3925	0.3174	1.51	0.8421
ExtraTrees	0.6806	0.1820	9.1974	4.1932	0.9292	4.28	0.6806
RandomForest	0.6543	0.2271	5.1995	1.1935	12.1927	4.53	0.6544
XGBoost	0.5724	0.2571	5.2001	10.1937	1.1930	4.72	0.5725
MLP	0.4846	0.4959	8.2100	8.1948	5.1938	5.20	0.5071
CatBoost	0.4426	0.1699	2.2031	3.1943	8.1933	6.13	0.4459
SVR	0.2714	0.2390	12.2081	2.1950	5.1937	7.60	0.2749

### 3.6 Feature importance and model interpretability

**Figure 6:**
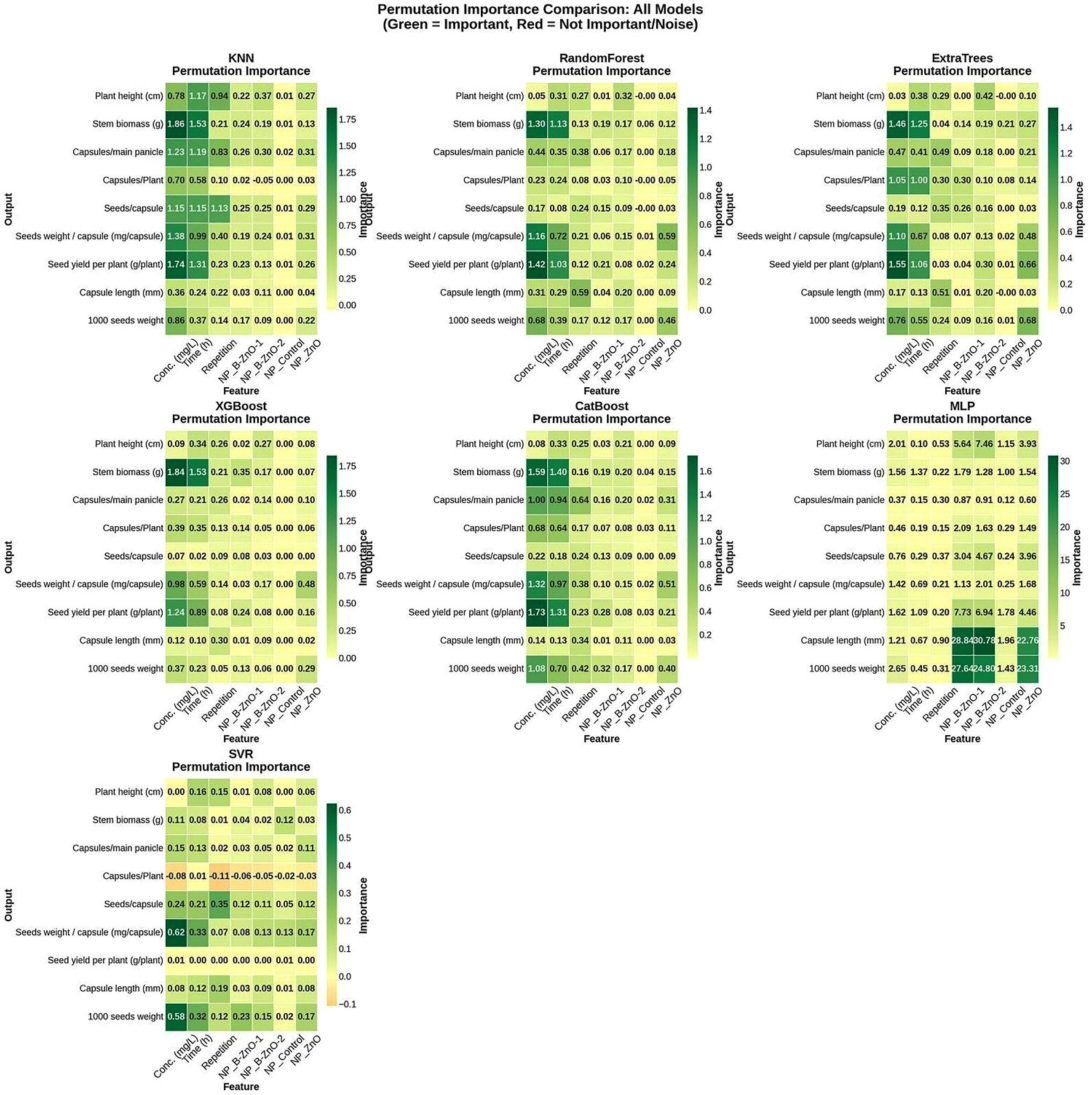
Heatmap comparison of permutation feature importance scores across seven machine learning models. Darker green indicates features that significantly influence the model’s prediction accuracy, while yellow/red indicates negligible impact.

A permutation Feature Importance analysis was conducted across all algorithms, presented in **Figure 6**, to interpret the decision-making logic of the Black Box models and identify the primary drivers of flax physiological traits. This method evaluates the decline in model performance when a specific feature’s values are randomly shuffled. From the analysis, concentration and ET are dominant predictors across the models, such as KNN and RF. For the KNN models, which had the highest R^2^ Score, the reliance on concentration and time suggests that the physiological responses of the plant are primarily governed by the dosage and duration of the NP exposure rather than the specific NP type. The behavior of MLP offers a different contrast that aligns with the performance metrics as represented in **Table 4**. While TB models ignored the categorical NP types, the MLP showed exceptionally high importance scores to specific NP identifiers when predicting CL. Conversely, the SVR exhibited uniformly low feature importance scores, reflecting its inability to establish strong decision boundaries. The variable Repetition showed how-to-moderate importance, confirming that while the experimental block design had some influence, it was not a confounding factor overshadowing the treatment effects.

**Table 4:** Recommended machine learning models for each specific output variable based on optimal statistical performance.

Output variable	Best R^2^ model	R^2^ score	Best MSE model	MSE score	Best MAE model	MAE score
Plant height (cm)	KNN	1.00	KNN	0.00	KNN	0.00
Stem biomass per plant (g)	KNN	1.00	KNN	0.00	KNN	0.00
Capsules/main panicle	KNN	1.00	KNN	0.00	KNN	0.00
Capsules/Plant	ExtraTrees	0.79	ExtraTrees	4.04	ExtraTrees	1.61
3.1 Seeds/capsule	3.2 KNN	3.3 1.00	3.4 KNN	3.5 0.00	3.6 KNN	3.7 0.00
Seeds weight/capsule (mg/capsule)	KNN	1.00	KNN	0.00	KNN	0.00
Seed yield per plant (g/plant)	KNN	1.00	KNN	0.00	KNN	0.00
Capsule length (mm)	MLP	0.77	MLP	0.02	MLP	0.12
1000 seeds weight	ExtraTrees	0.72	ExtraTrees	0.01	ExtraTrees	0.09

## 4 Discussion

Results of ZnO nanoparticle priming revealed that agronomic traits are governed by the balanced interaction between physicochemical properties of NPs, concentrations, and priming time (Donia and Carbone, 2023). Results revealed superior performance of biologically synthesized ZnO NPs compared to the chemical-based NPs and the control (Coppa et al., 2024). The superiority suggests that surface chemistry and particle methodology may enhance interaction with the seed coat and embryonic tissues (Pandya et al., 2024). The controlled release of organic functional groups may regulate redox balance during early germination, promoting cell division and elongation without inducing oxidative stress (Donia and Carbone, 2023; Pandya et al., 2024), as confirmed by a better response than control while considering all agronomic traits. Results further illustrated the concentration-dependent pattern, and moderate dosage was found to be optimum for stimulating growth, while excessive concentration induced toxicity on some traits, supporting the concept of hormesis (Gao et al., 2025). These findings are in agreement with recent work on Pennisetum glaucum, where green-synthesized ZnO nanoparticles applied at 100–150 mg kg^-^¹ significantly enhanced germination, chlorophyll content, antioxidant activity, and transcription factor expression, whereas higher concentrations induced inhibitory effects, further supporting the hormetic response and dose-dependent behavior of ZnO NPs (Shivhare et al., 2025). The negative impact caused by high concentrations might be due to overproduction of ROS, membrane damage, or interference with metabolic processes (Pandya et al., 2024). Likewise, exposure time is crucial as it regulates the adsorption followed by absorption of NPs into seed tissues (Donia and Carbone, 2023). The Zn uptake dynamics and early metabolic activation are regulated by exposure time, and prolonged exposure may lead to increased cellular accumulation and modulation of transcriptional responses related to growth and development (Geremew et al., 2025).

The results of individual factors associated with their interaction modulated most of the agronomic traits (Ravishankar et al., 2025). Results revealed that interaction-dependent enhancement of yield-related traits influences not only vegetative growth but also sink strength, assimilate partitioning, and reproductive efficiency (Waqas et al., 2022). Results revealed that the interaction of NPs type, concentration, and priming time variable treatment time for agronomic traits, and biogenic ZnO NPs was found to be optimum for most of the agronomic traits (Coppa et al., 2024). Among the parameters, 25 mg concentration was the most optimal concentration for seed yield for all types of NPs. The overall results support the findings that ZnO nano-priming acts as a regulatory stimulus rather than merely a nutrient supplement, and performs better than control treatments (Donia and Carbone, 2023; Waqas et al., 2022).

RSM is a statistical tool designed for optimizing multiple integrating variables, especially complex biological systems (Silva and Cardoso, 2024). It allows researchers to identify the significant factors, magnitude, and direction of interactions among variables that may not be detectable through conventional statistical approaches (Bulut et al., 2025). Results of RSM-based ANOVA analysis revealed the model’s robustness for certain agronomic traits, indicating that the fitted polynomial relationship adequately presents the true response surface within the experimental region (Ruby-Figueroa, 2015). The presence of both linear and quadratic significance confirms that the biological response involves curvature and interaction effects (Katirci et al., 2021). Therefore, results were further computed with the Pareto charts and normal plots for a better understanding of the responses. The Pareto chart is a diagnostic tool used in DOE to visualize the magnitude and statistical significance of standardized effects (Larson and Whitney, 2025). It also ranks the effects by stacking them from largest to smallest, and uses a reference line to determine the significance, and also a threshold (Aasim et al., 2026). The results of Pareto charts revealed that each trait exhibited a unique stacking order of predictor importance, indicating the variability due to physiological or environmental manipulations. Results further revealed the significance of NPs type (C factor) and the interaction of the two factors modulates the morphological and reproductive responses. In the further step, the direction (positive or negative) and magnitude of each predictor were examined through the normal plot, placing them on the right (positive) or left (negative) side of the standard line (Aasim et al., 2023). Results illustrated that most of the predictors were placed on the right side, suggesting the proportional (positive) influence on the responses. However, the negative impact of predictors was also noted, indicating the concentration-induced physiological stress for the number of capsules and seeds per capsule. Overall results indicate that most of the factors were influenced by at least one predictor, and some traits were statistically insignificant, demonstrating the need for further optimization.

The Pearson correlation analysis revealed clear patterns for the measured traits, indicating both positive and negative relationships. The strong positive correlation of SYP and WP with CP indicates that reproductive output and vegetative growth are the major contributors to seed yield, confirming the significance of capsules and seeds per capsule for seed yield (Parameswari et al., 2019). Whereas a moderate positive correlation between SWC and SC implies that capsule producing more seeds tend to exhibit higher seed yield (Mitkari et al., 2023). Conversely, the negative correlation between NPs concentration and seed-related traits (SWC, SYP) suggests that higher nutrient levels may induce physiological stress, disrupt plant growth, and reproductive performance (Pandya et al., 2024). Overall analysis revealed interdependencies among the variables and justifies the need for regression-based ML models for better prediction in plant breeding studies (Parveen et al., 2025).

The predictive evaluation of nine physiological and morphological traits revealed the variable performance of ML models, a phenomenon very common in plant sciences (Martinović et al., 2025; Aasim et al., 2026). Such variability is well documented in biological datasets, where nonlinear interactions, multicollinearity among traits, and limited sample size often challenge global learning models and favor locally adaptive or ensemble-based approaches (John et al., 2022). The KNN model achieved the highest accuracy with a low MSE score, indicating that the local instance-based model captures the nonlinear and multivariate relationship between NPs and flax traits. Results confirmed the previous work, where the KNN model outperformed more complex architectures when trait-response patterns exhibit strong local structure or heterogeneous variance (Gill et al., 2022). Instance-based learners such as KNN are particularly effective in agronomic experiments with factorial designs and discrete treatment levels, as they rely on neighborhood similarity rather than strong assumptions (Assis et al., 2024).

The ET and RF models were ranked next for prediction, indicating the consistent and known strength of ensemble tree algorithms to capture complex trait interactions through hierarchical feature positioning (Maturo and Riccio; Tosan et al., 2025). These models effectively handle noise and multicollinearity and remain reliable when nonlinear interactions shape plant trait responses (Kozlov et al., 2025; Tosan et al., 2025). Results further revealed reasonable predictive performance of XGBoost, MLP, and CatBoost models. Gradient-boosted decision tree models are generally reliable for agronomic traits prediction; their performance varies with dataset, noise structure, and hyperparameter optimization (Gill et al., 2022). The moderate performance of the MLP model was also attributed to an insufficient sample size for stable generalization as neural network models require larger and more diverse datasets to fully exploit their representational capacity in biological systems (Ursu et al., 2025). The lowest performance of the SVR model suggests that default kernel configurations failed to capture the nonlinear and interacting effect of NPs on agronomic traits of flax (Kozlov et al., 2025). This highlights the sensitivity of kernel-based choice and parameter tuning, which can limit their practical use in exploratory agronomic modeling without extensive optimization (Burglewski et al., 2025; Singh and Walia, 2025). Overall results demonstrate that algorithms capable of leveraging localized structure or ensemble-based nonlinear partitioning are better suited for complex physiological responses elicited by the NPs treatments. Conversely, models requiring specialized kernel or architectural tuning performed poorly, underscoring the significance of algorithm-data compatibility.

The heatmap analysis of traits with ML models is used to identify which plant traits are inherently more predictable and which require complex modeling (Wang et al., 2022), a practice recommended for modern ML-based phenotyping frameworks (John et al., 2022). Such visualization tools also facilitate model interpretability and help prioritize traits that are more amenable to predictive breeding or precision input management (Danilevicz et al., 2025). Results indicated that the KNN model performed strongly for PH, SBP, and SYP, while CP remained poorly predicted, confirming the consistent pattern for trait complexity and environmental sensitivity (Kaur and Paul, 2020). The predictive reliability of models was further assessed by correlation analysis of error metrics and models. The KNN model maintained the lowest error metrics, indicating that its local-averaging mechanism effectively minimizes both systematic and absolute deviations. In contrast, MLP and SVR models computed higher MSE scores, reflecting greater variability and poorer fit under the nonlinear conditions.

The permutation feature-importance analysis provides valuable insight into how different ML models prioritize the underlying biological signals (Biswas et al., 2025; Huang, 2025). The consistent dominance of concentration and exposure time for all models aligns with the well-known dose-response dynamics in NP-plant interaction (Pandya et al., 2024). This convergence across algorithmic families suggests that these variables represent fundamental drivers of stress modulation and trait expression rather than model-specific artifacts. The contrasting behaviors of the MLP model further underscore the role of model structure in shaping interpretability (Ursu et al., 2025). The uniform weak performance of the SVR model reflects its limited capacity to generalize in high-dimensional and nonlinear spaces (Burglewski et al., 2025). The moderate signal from the repetition factor implies that block effects were present but not dominant (Katırcı et al., 2021). These results confirm that treatment responses were driving the predictive patterns rather than the experimental layout.

## 5 Conclusion

Green-synthesized ZnO nanoparticles used as seed-priming agents significantly improved linseed growth and yield traits compared with chemically synthesized nanoparticles and bulk ZnO, highlighting the importance of nanoparticle origin and surface chemistry. Plant responses exhibited a dose-time dependency consistent with hormesis: moderate concentrations enhanced performance, whereas higher levels reduced seed-related traits, indicating the need for careful optimization. DOE/RSM analyzes confirmed that nanoparticle type, concentration, and priming duration interactively influence phenotypes, emphasizing that single-factor approaches are insufficient for nanoparticle-based interventions. Machine learning models captured complex nonlinear relationships among inputs and multiple correlated outputs, with K-Nearest Neighbors (KNN) achieving the highest predictive accuracy. Feature importance analysis identified nanoparticle concentration and exposure time as the primary drivers of plant responses. These findings suggest that biogenic ZnO nanopriming can serve as an efficient strategy for micronutrient delivery and early seedling vigor, provided concentration and exposure are carefully tuned. Future work should validate optimal settings across cultivars, seasons, and soil types; elucidate mechanistic links through Zn uptake, antioxidant responses, and metabolic profiling; assess nanoparticle fate and environmental safety; and enhance predictive modeling through external validation and sensitivity analysis. Overall, integrating green nanomaterials with DOE optimization and interpretable machine learning provides a scalable framework for precision seed enhancement and sustainable linseed production.

## Data Availability

The original contributions presented in the study are included in the article/**Supplementary Material**. Further inquiries can be directed to the corresponding author.
