# Supplementary material for: Machine learning and mathematical modeling for comparative analysis of green-synthesized ZnO nanoparticles as seed nano-priming agents for linseed
Source: Front Plant Sci. 2026 May 11;17:1827745. doi: 10.3389/fpls.2026.1827745 (PMC13199122; doi:10.3389/fpls.2026.1827745)
Supplement: Supplementary file 1 [file Table1.docx]

Table S1: One-Way ANOVA analysis of nanopriming impact of different types of ZnONPs on agronomic traits of linseed

| **ZnO NPs** | | | | | | | | | |
| --- | --- | --- | --- | --- | --- | --- | --- | --- | --- |
| **Chemıcal** | **Plant height (cm)**** | **Technical weight/plant (g)*** | **No. of capsules ın the maın** | **No.of capsules / plant** | **No. of seeds/ capsule*** | **Seeds per capsule** | **Seed yield g/plant)** | **Capsule Length (mm)** | **1000 seeds weight** |
| **Control** | 74.63±2.91ab | 1.927±0.027a* | 23.867±1.504 | 33.200±1.015 | 7.967±0.416ab | 39.82±2.15 | 1.174±0.096 | 7.223±0.357 | 5.565±0.045 |
| **ZnO** | 71.893±4.261b | 1.513±0.196b | 20.744±2.828 | 28.989±3.643 | 8.133±0.688ab | 45.38±7.98 | 1.013±0.156 | 7.199±0.341 | 5.398±0.199 |
| **B-Zno-1** | 73.367±3.791ab | 1.589±0.270ab | 21.952±3.358 | 30.478±4.568 | 7.711±0.648b | 42.821±3.94 | 1.086±0.164 | 7.207±0.327 | 5.557±0.237 |
| **B-Zno-2** | 76.25±5.20a** | 1.591±0.226ab | 22.007±3.187 | 29.00±5.24 | 8.241±0.829a* | 45.353±4.32 | 1.013±0.156 | 7.324±0.305 | 5.568±0.247 |
| **Concentration** | | | | | | | | | |
| **Conc.** | **Plant height (cm)** | **Technical weight / plant (g)** | **No. of capsules ın the maın** | **No.of capsules / plant** | **No. of seeds/ capsule** | **Seeds per capsule** | **Seed yield**  **g/plant** | **Capsule Length (mm)** | **1000 seeds weight** |
| **0** | 74.63±2.91 | 1.927±0.027 | 23.867±1.504 | 33.20±1.015 | 7.967±0.416 | 39.82±2.15b | 1.174±0.095 | 7.223±0.35 | 5.565±0.047 |
| **25** | 73.27±5.85 | 1.549±0.266 | 21.463±3.563 | 28.99±5.36 | 8.304±0.821 | 48.44±6.04a** | 1.066±0.164 | 7.206±0.351 | 5.570±0.265 |
| **50** | 74.88±3.829 | 1.541±0.200 | 22.559±2.769 | 29.89±4.676 | 7.937±0.575 | 43.71±4.42b | 1.030±0.181 | 7.164±0.306 | 5.482±0.214 |
| **75** | 73.35±4.379 | 1.601±0.231 | 20.681±2.877 | 29.58±3.476 | 7.844±0.785 | 41.42±4.44b | 1.039±0.130 | 7.358±0.298 | 5.473±0.234 |
| **Treatment Time** | | | | | | | | | |
| **Time (h)** | **Plant height (cm)** ** | **Technical weight / plant (g)** | **No. of capsules ın the maın** | **No.of capsules / plant** | **No. of seeds/ capsule** | **Seeds per capsule** | **Seed yield g/plant** | **Capsule Length (mm)** | **1000 seeds weight** |
| **1** | 71.700±3.493b | 1.5841±0.191 | 20.989±3.469 | 29.986±4.163 | 8.079±0.745 | 45.60±6.44 | 1.078±0.131 | 7.210±0.345 | 5.503±0.216 |
| **2** | 74.346±4.519ab | 1.6110±0.315 | 22.132±3.138 | 30.479±5.098 | 7.939±0.762 | 43.69±5.77 | 1.032±0.206 | 7.281±0.259 | 5.482±0.215 |
| **4** | 75.550±5.216a | 1.5364±0.189 | 21.829±2.736 | 28.396±4.065 | 8.061±0.738 | 43.76±4.907 | 1.039±0.127 | 7.237±0.366 | 5.545±0.273 |

Table S2: RSM-based ANOVA analysis of ZnO nanoparticles on agronomic traits of linseed

| **Source** | **Plant height (cm)** | **Technical weight / plant (g)** | **No. of capsules ın the maın** | **No.of capsules / plant** | **No. of seeds/ capsule** | **Amount of seeds in capsule** | **Seed yield g/plant)** | **Capsule Length (mm)** | **1000 seeds weight**** |
| --- | --- | --- | --- | --- | --- | --- | --- | --- | --- |
| **Model** | 0.003** | 0.008** | 0.080 | 0.094 | 0.022* | 0.000** | 0.000** | 0.048* | 0.040* |
| **Linear** | 0.001** | 0.176 | 0.206 | 0.520 | 0.047* | 0.003** | 0.131 | 0.371 | 0.050 |
| **Conc^. (mg/L)^** | 0.154 | 0.891 | 0.178 | 0.438 | 0.051 | 0.000** | 0.317 | 0.613 | 0.143 |
| **Time ^(h)^** | 0.041* | 0.721 | 0.738 | 0.775 | 0.817 | 0.834 | 0.477 | 0.242 | 0.803 |
| **NP** | 0.001** | 0.246 | 0.060 | 0.240 | 0.015* | 0.001** | 0.193 | 0.314 | 0.017* |
| **Conc.^mg/L^*Conc.^mg/L^** | 0.109 | 0.504 | 0.036* | 0.545 | 0.395 | 0.249 | 0.472 | 0.103 | 0.447 |
| **2-Way Interaction** | 0.383 | 0.011* | 0.161 | 0.067 | 0.094 | 0.009** | 0.000** | 0.039 | 0.123 |
| **Conc. ^mg/L^*Time  ^(h)^** | 0.948 | 0.000** | 0.142 | 0.003** | 0.347 | 0.249 | 0.000** | 0.246 | 0.184 |
| **Time  ^(h)*^NP** | 0.216 | 0.671 | 0.156 | 0.425 | 0.049 | 0.007** | 0.046* | 0.031* | 0.142 |
