# Supplementary material for: Machine learning and mathematical modeling for comparative analysis of green-synthesized ZnO nanoparticles as seed nano-priming agents for linseed
Source: Front Plant Sci. 2026 May 11;17:1827745. doi: 10.3389/fpls.2026.1827745 (PMC13199122; doi:10.3389/fpls.2026.1827745)
Supplement: Supplementary file 2 [file DataSheet1.docx]

**Characterization of Nanoparticles**


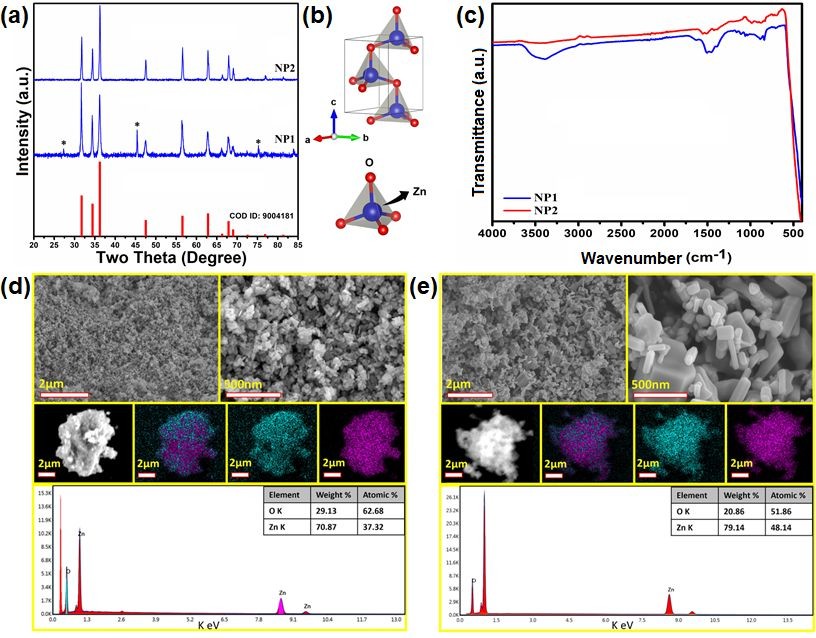
The crystallographic properties of the ZnO samples were characterized using a Bruker D8 Advance X-ray diffractometer (Cu Kα, λ = 1.5406 Å). Surface morphology of both the commercial and biogenic nanoparticles was analyzed by FE-SEM (HITACHI SU5000, Japan) coupled with an EDX detector for elemental composition. FT-IR measurements were carried out using a Bruker Vertex ATR-FTIR spectrophotometer (Bruker Optics, USA) to identify the functional groups present on the nanoparticle surfaces.

**Supplementary Figure S1.** (a) XRD patterns of biogenic (NP1) and commercial (NP2) ZnO samples, confirming the hexagonal wurtzite crystal structure. (b) Three-dimensional schematic representation of the wurtzite ZnO crystal structure. (c) FTIR spectra of NP1 and NP2 demonstrating the characteristic Zn–O stretching vibrations along with surface functional groups. (d) SEM micrographs, elemental mapping, and EDX spectrum of biogenic ZnO (NP1), showing agglomerated nanoscale particles and the elemental distribution of Zn and O. (e) SEM images, elemental mapping, and EDX profile of commercial ZnO (NP2), indicating well-defined particle morphology and homogeneous elemental composition.
